# Supplementary material for: Nanoemulsions of Jasminum humile L. and Jasminum grandiflorum L. Essential Oils: An Approach to Enhance Their Cytotoxic and Antiviral Effects
Source: Molecules. 2022 Jun 6;27(11):3639. doi: 10.3390/molecules27113639 (PMC9182578; doi:10.3390/molecules27113639)
Supplement: Supplementary file 1 [file molecules-27-03639-s001.zip › molecules-1723655-supplementary.pdf]

Nanoemulsions of *Jasminum humile* L. and *Jasminum grandiflorum* L. essential oils: an approach to enhance their cytotoxic and antiviral effects

Khaled Ahmed Mansour[a, b], Mona El-Neketi[a, *], Mohamed-Farid Lahloub[a], Ahmed Elbermawi[a, *]

[a] Department of Pharmacognosy, Faculty of Pharmacy, Mansoura University, 35516 Mansoura, Egypt

[b] Department of Pharmacognosy, Faculty of Pharmacy, Horus University in Egypt, 34517 New Damietta, Egypt

E-mail address: Khaled.mansour@o6u.edu.eg; Khali@horus.edu.eg (K.A. Mansour)

E-mail address: melneketi@mans.edu.eg (M. El-Neketi), monaneketi@gmail.com

E-mail address: mfil@mans.edu.eg (M.F. Lahloub)

E-mail address: Asbeder@mans.edu.eg (A. Elbermawi)

[*]Corresponding author at: Department of Pharmacognosy, Faculty of Pharmacy, Mansoura University, 35516 Mansoura, Egypt

Telephone:

+20-011-57-557-280

E-mail address: melneketi@mans.edu.eg (M. El-Neketi)

+20-010-04-811-533

E-mail address: Asbeder@mans.edu.eg (A. Elbermawi)

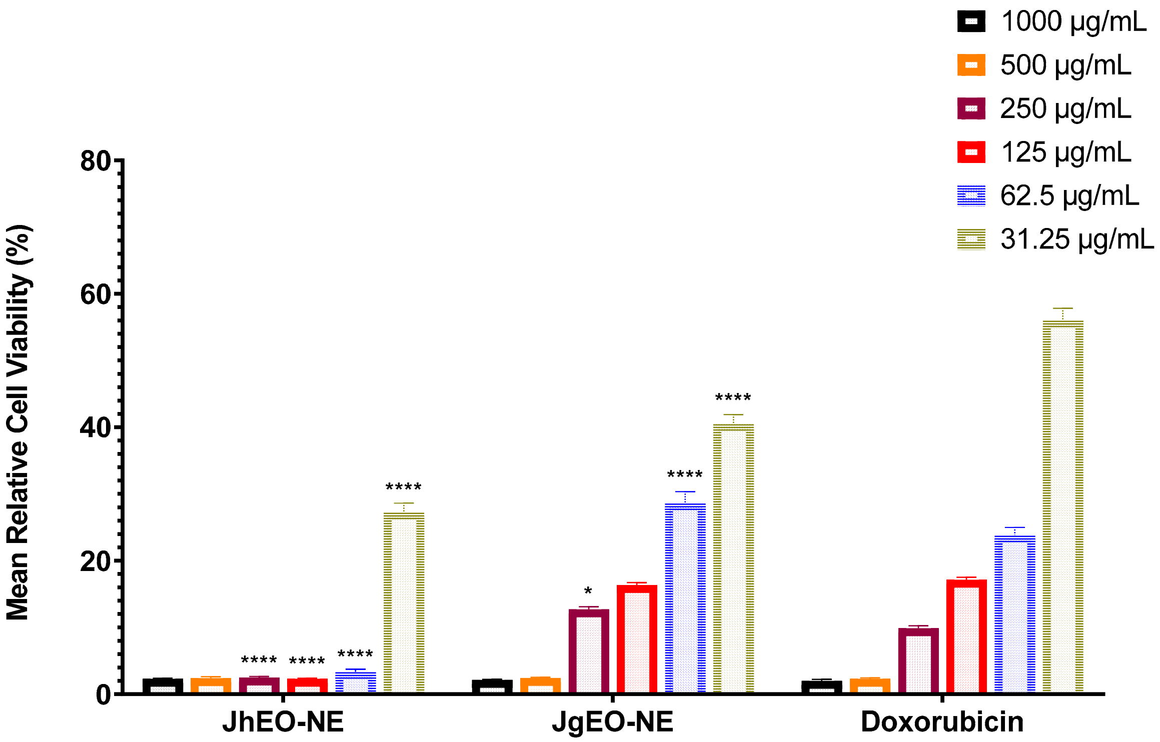

Figure S1. Cytotoxic activity of various concentrations the nanoemulsion formulations of the essential oils obtained from *Jasminum humile* L. (JhEO-NE) and *Jasminum grandiflorum* L. (JgEO-NE) as well as standard doxorubicin against the leukemia cell line, HepG-2. Bar graphs represent the mean ± SEM of 3 determinations. Asterisks show statistical significance ($p \leq 0.05$) compared to the reference drug doxorubicin. (*) denotes $p \leq 0.05$. (**) denotes $p \leq 0.01$. (***) denotes $p \leq 0.001$. (****) denotes $p \leq 0.0001$.

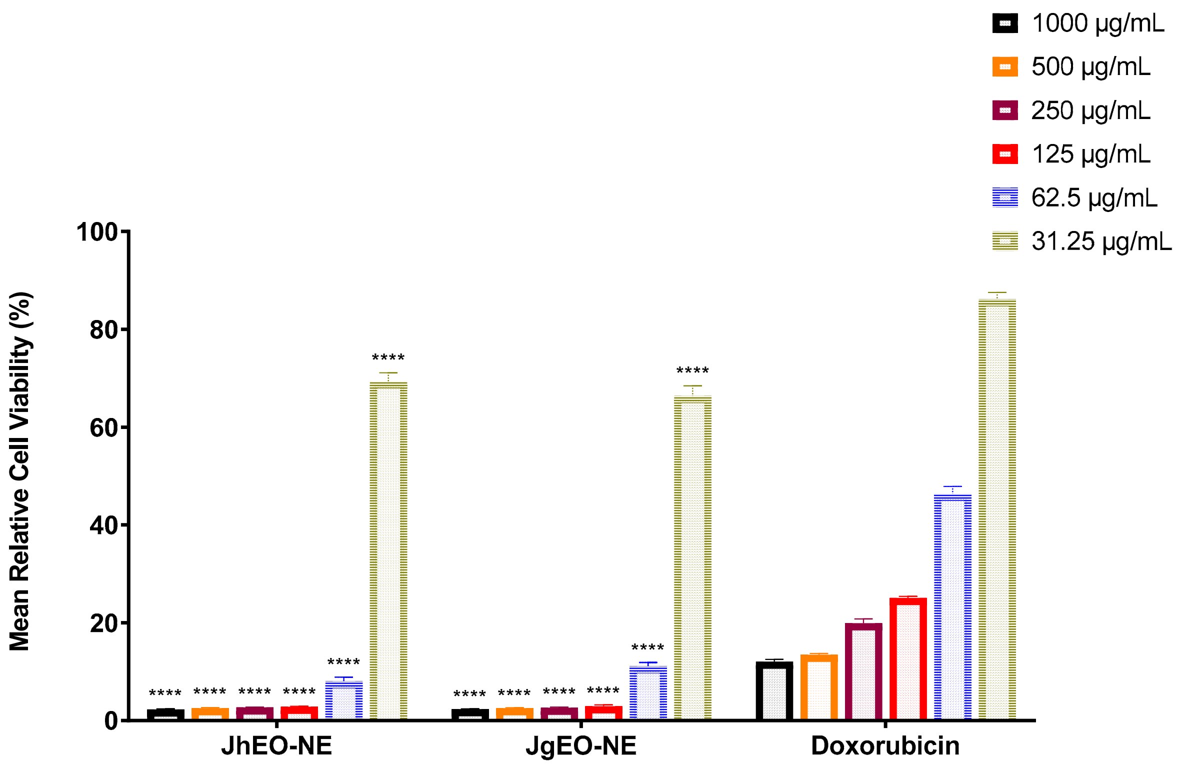

Figure S2. Cytotoxic activity of various concentrations of the nanoemulsion formulations of the essential oils obtained from *Jasminum humile* L. (JhEO-NE) and *Jasminum grandiflorum* L. (JgEO-NE) as well as standard doxorubicin against the leukemia cell line, MCF-7. Bar graphs represent the mean ± SEM of 3 determinations. Asterisks show statistical significance ($p \leq 0.05$) compared to the reference drug doxorubicin. (*) denotes $p \leq 0.05$. (**) denotes $p \leq 0.01$. (***) denotes $p \leq 0.001$. (****) denotes $p \leq 0.0001$.

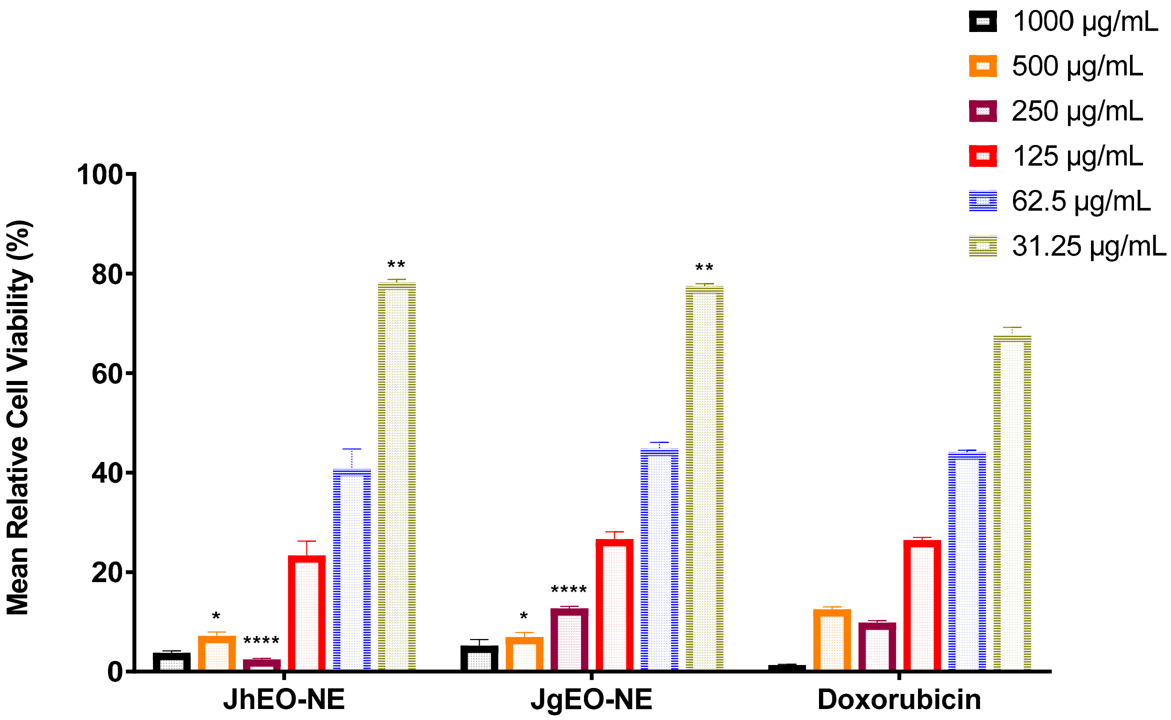

Figure S3. Cytotoxic activity of various concentrations of the nanoemulsion formulations of the essential oils obtained from *Jasminum humile* L. (JhEO-NE) and *Jasminum grandiflorum* L. (JgEO-NE) as well as standard doxorubicin against the leukemia cell line, THP-1. Bar graphs represent the mean ± SEM of 3 determinations. Asterisks show statistical significance ($p \leq 0.05$) compared to the reference drug doxorubicin. (*) denotes $p \leq 0.05$. (**) denotes $p \leq 0.01$. (***) denotes $p \leq 0.001$. (****) denotes $p \leq 0.0001$.

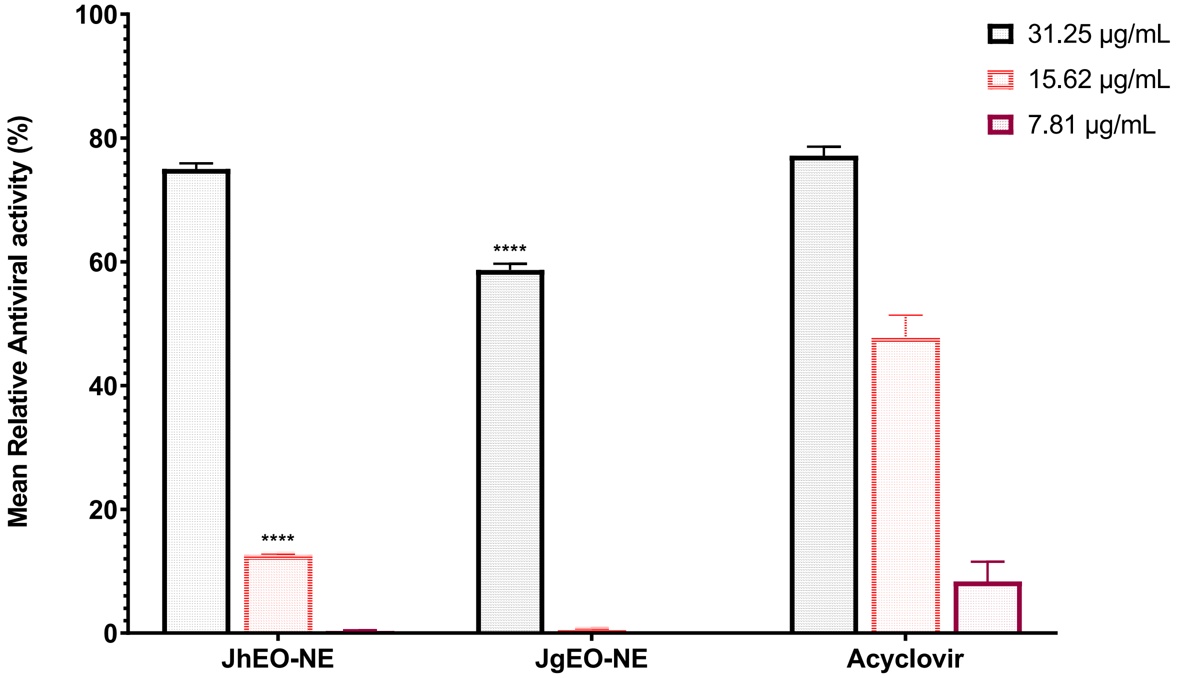

Figure S4. Antiviral activity of various concentrations of the nanoemulsion formulations of the essential oils obtained from *Jasminum humile* L. (JhEO-NE) and *Jasminum grandiflorum* L. (JgEO-NE) as well as standard doxorubicin against HAV virus. Bar graphs represent the mean ± SEM of 3 determinations. Asterisks show statistical significance ($p \leq 0.05$) compared to the reference drug doxorubicin. (*) denotes $p \leq 0.05$. (**) denotes $p \leq 0.01$. (***) denotes $p \leq 0.001$. (****) denotes $p \leq 0.0001$.

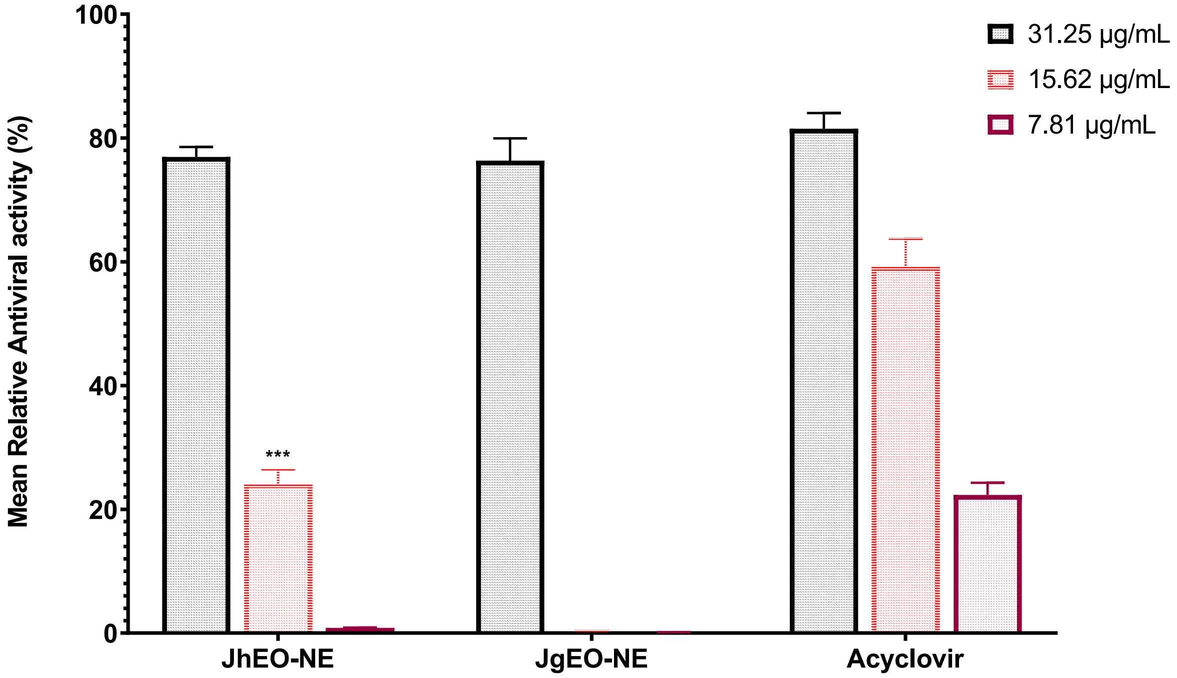

Figure S5. Antiviral activity of various concentrations of the nanoemulsion formulations of the essential oils obtained from *Jasminum humile* L. (JhEO-NE) and *Jasminum grandiflorum* L. (JgEO-NE) as well as standard doxorubicin against HSV-1 virus. Bar graphs represent the mean ± SEM of 3 determinations. Asterisks show statistical significance ($p \leq 0.05$) compared to the reference drug doxorubicin. (*) denotes $p \leq 0.05$. (**) denotes $p \leq 0.01$. (***) denotes $p \leq 0.001$. (****) denotes $p \leq 0.0001$.
